# Nonsurgical Rehabilitation in Dachshunds With T3-L3 Myelopathy: Prognosis and Rates of Recurrence

**DOI:** 10.3389/fvets.2022.934789

**Published:** 2022-07-19

**Authors:** Jordan Sedlacek, Jessica Rychel, Michelle Giuffrida, Bonnie Wright

**Affiliations:** ^1^Fort Collins Veterinary Emergency and Rehabilitation Hospital, Fort Collins, CO, United States; ^2^Red Sage Integrative Veterinary Partners, Fort Collins, CO, United States; ^3^Department of Surgical and Radiological Sciences, School of Veterinary Medicine, University of California, Davis, Davis, CA, United States; ^4^MistralVet, Johnstown, CO, United States

**Keywords:** rehabilitation, T3-L3 myelopathy, veterinary neurology, acupuncture, nonsurgical, hyperbaric oxygen, IVDH

## Abstract

Dachshunds are at significant risk of experiencing thoracolumbar intervertebral disk herniation (IVDH) during their lifetimes. Standard of care includes advanced imaging, surgical intervention, and postoperative rehabilitation. Conservative management is commonly recommended for cases where the standard of care is declined, and little is known about the prognosis of treatment with conservative management and rehabilitation (nonsurgical rehabilitation). This retrospective cohort study assessed 12-week functional outcome and recurrence of clinical signs in 40 dachshunds with T3-L3 myelopathy presumed to be due to Hansen's Type I disc herniation, treated with nonsurgical rehabilitation. The overall prognosis was good with 34 of 40 (85.0%, 95% CI 70.2–94.2) dachshunds achieving functional pet status by 12 weeks postinjury. Modified Frankel Score at presentation was significantly (*p* < 0.001) higher in dogs with a positive 12-week outcome compared to dogs that did not recover by 12 weeks. All 27 dogs with motor function at presentation had a positive outcome. Of the 9 dogs exhibiting paraplegia with intact deep nociception at presentation, 7 dogs (77.8%) had achieved a positive outcome by 12 weeks. None of the 4 dogs persistently lacking deep nociception had a positive outcome. Among 27 dogs with a positive outcome for whom follow-up records were available, the 1- and 2-year recurrence rates for T3-L3 myelopathy were 5 and 11%, respectively. Nonsurgical rehabilitation should be considered in dachshunds with mild to moderate T3-L3 myelopathy or in severe cases when advanced imaging and surgical intervention are not possible.

## Introduction

Intervertebral disk herniation (IVDH) is a common condition in dogs with a lifetime prevalence of ~3.5%. The incidence is significantly higher in chondrodysplastic breeds with a 20% lifetime prevalence in the miniature dachshund (1). These injuries most commonly occur in the thoracolumbar (T3-L3) region of the spine (2–4). The current standard of care for T3-L3 myelopathy with severe neurologic signs or refractory pain recommends advanced imaging to identify the level of injury and diagnose the underlying etiology, followed by surgical intervention to address the primary injury to the spinal cord when possible (5). Studies have found that dogs with IVDH that undergo hemilaminectomy have a good prognosis for return to normal function if deep nociception is present prior to surgery (97.7% return to ambulation). If deep nociception is not present prior to surgery the likelihood of return to ambulation decreases (52.1%) (6). Less severe injuries are associated with earlier time to ambulation in the postoperative period (6, 7).

In a review from 2016, rehabilitation was recommended after hemilaminectomy by 64% of board-certified surgeons, and 46% of board-certified neurologists (8). Rehabilitation typically consists of a variety of techniques and modalities including, but not limited to, passive range of motion, therapeutic exercise, underwater treadmill, manual therapy, acupuncture or electroacupuncture, photobiomodulation, transcutaneous electrical nerve stimulation (TENS), neuromuscular electrical stimulation (NMES), pulsed electromagnetic field therapy (PEMF), and hyperbaric oxygen therapy (HBOT) (9–12). A retrospective analysis of postoperative rehabilitation following T3-L3 hemilaminectomy in 2015 found an association between rehabilitation and positive outcomes after surgery (13). Rehabilitation has also been associated with more complete recovery after surgery (14, 15). However, recent prospective studies have had more equivocal results (16, 17).

Due to financial constraints or personal preferences, many owners are unable to pursue the standard of care detailed above. For these patients, medical management is typically recommended. Usually, this includes cage rest, pharmaceutical administration (analgesics, muscle relaxants, and anti-inflammatory medications), and if possible physical rehabilitation. Levine et al. found that in 223 dogs with presumed thoracolumbar IVDH receiving cage rest and medications, 54.4% achieved a successful outcome defined as a significant improvement in neurologic function with no report of recurrence of clinical signs. An additional 14.5% were considered treatment failures, progressing to surgery, or euthanasia. Dogs undergoing conservative management exhibited rates of recurrence similar to surgically treated dogs with thoracolumbar disc herniations. Successful outcomes were found to be associated with the duration of clinical signs at admission. The study did not assess participation in a rehabilitation program as a parameter of conservative management (4).

The first study that described nonsurgical rehabilitation techniques for T3-L3 myelopathy in dogs was published by Jadeson in 1961. Eighty-two dogs were treated for their myelopathy with conventional treatment including nursing care, muscle relaxants, steroids, antibiotics, vitamin B complex, and vitamin D. Forty-seven of the group received rehabilitation sessions in addition to conventional treatment. Rehabilitation techniques included hydrotherapy, massage, heat treatments, manual therapy, and assistive devices such as carts. The study found that a higher proportion of dogs received a good or excellent grade of improvement when rehabilitation was included in the treatment plan with greater differences noted in more severe injuries. Overall, 89.36% of dogs in the rehabilitation group had a good or excellent improvement compared to 77.14% of dogs in the conventional treatment group. Among dogs with paralysis, the rates drop to 88.46 and 72.22%, respectively (18).

The purpose of this retrospective study is to assess the outcome for dachshunds exhibiting presumed thoracolumbar IVDH who undergo nonsurgical rehabilitation. For patients not pursuing surgery, advanced imaging is often not undertaken due to either expense or feasibility. For many of these cases, a diagnosis of T3-L3 myelopathy with presumed Hansen Type I IVDH is made *via* signalment, physical exam findings, and neuroanatomic localization alone.

The primary objective of this study is to assess the likelihood of a positive case outcome in dachshunds with presumed thoracolumbar Hansen Type I IVDH treated with nonsurgical rehabilitation given the severity of neurologic signs at presentation. The primary hypothesis is that in dachshunds treated with nonsurgical rehabilitation, a positive functional outcome at 12 weeks is associated with the severity of neurologic signs at presentation as defined by a Modified Frankel Score (MFS) (19). A secondary objective is to describe the incidence and timing of recurrent T3-L3 myelopathy in dogs that are successfully treated with rehabilitation by 12 weeks postinjury.

## Materials and Methods

This study was conducted as a retrospective analysis of medical records from the Fort Collins Veterinary Emergency and Rehabilitation Hospital from January 2010 through October 2020. The electronic medical record database was searched for all records of dachshunds and dachshund mixed breed dogs that presented to the hospital during the study period. These were then screened to include only dachshunds who had at least one visit to the rehabilitation department. The remaining medical records were then read and assessed by a rehabilitation-certified veterinarian (JS) to verify if cases met the selection criteria. Records were selected for inclusion in the study if the animal presented to the rehabilitation service and was diagnosed with T3-L3 myelopathy prior to the visit or at the initial evaluation. Clinical signs must have been present for 30 days or less before presentation to the rehabilitation service. Dogs who received care in the acute phase of injury by other departments or facilities were not excluded from the analysis. The patient must have completed a full initial evaluation with the rehabilitation service to be included. Exclusion criteria included: chronic cases with signs lasting for more than 30 days prior to presentation, history of hemilaminectomy or other spinal surgery, multifocal neurologic disease (e.g., history of concurrent cervical or lumbosacral injury), or incomplete medical records.

Medical records were evaluated for sex, age, and MFS at presentation as defined in Table 1, as well as outcome 12 weeks after presentation. A positive outcome was defined as an animal who had regained the ability to be a functional pet: urinary and fecal continence, the ability to ambulate without assistance, absent to minimal neurologic deficits (mild proprioceptive deficits in hindlimbs acceptable), and satisfactory pain management such that owner reports no limitations on the dog's quality of life (as determined through visit history forms).

**Table 1:** Modified Frankel Score.

**MFS grade**	**Physical exam findings**
Grade 0	Paraplegia with no deep nociception
Grade 1	Paraplegia with no superficial nociception
Grade 2	Paraplegia with nociception
Grade 3b	Non-weight bearing non-ambulatory paraparesis
Grade 3a	Weight bearing non-ambulatory paraparesis
Grade 4	Paraparesis and ataxia
Grade 5	Spinal hyperesthesia only

*Source: Levine et al. (19)*.

All dogs underwent rehabilitation under the guidance of a veterinarian certified in canine rehabilitation or board-certified in canine rehabilitation and sports medicine. Individual protocols varied based on patient needs and owner availability. In general, rehabilitation protocols included a combination of the following: therapeutic exercise, gait training (underwater treadmill or supported land ambulation), photobiomodulation, manual therapy, acupuncture/electroacupuncture, TENS, NMES, and PEMF. Patients were prescribed medications as needed to manage pain, inflammation, and urinary bladder dysfunction. Most patients were prescribed a home exercise plan to be completed by the owners. Some patients received over-the-counter supplements including fish oil and curcumin. Additionally, some patients received HBOT.

In order to assess the likelihood of recurrence, the length of follow-up after 12 weeks was recorded for all cases with successful treatment outcomes. A recurrence was defined as a loss of functional pet status as defined above, e.g., loss of unsupported ambulation, spinal hyperesthesia, loss of continence, etc.

Statistical analysis was performed with computer software.^1^ The population rate of positive outcome was estimated with an exact binomial confidence interval. Wilcoxon rank-sum test was used to compare MFS on a 0–5 scale according to a positive outcome at 12 weeks as defined above. Life table methods were used to estimate 1- and 2-year recurrence rates among dogs with positive outcomes at 12 weeks; dogs that died or were lost to follow-up without experiencing recurrence and dogs that were alive without recurrence at the time of data collection were censored at their last known live dates. Tests were 2-sided and *p* < 0.05 was statistically significant.

## Results

A total of 49 cases met the criteria for inclusion in the study. Of these, 23 were neutered males, 23 were spayed females, 2 were intact males, and 1 was an intact female. The median age at presentation was 7 years (Range: 3–15 years). The median duration of signs prior to presentation to the rehabilitation service was 3 days (Range: 1–30 days). Of the 49 cases, 9 were lost to follow-up during the study period.

For the 40 dogs with complete records, at the time of initial evaluation by the rehabilitation service, 4 dogs were lacking deep nociception (MFS = 0), 4 dogs had deep nociception but were lacking superficial nociception (MFS = 1), 5 dogs exhibited paraplegia with nociception (MFS = 2), 7 dogs exhibited nonweight bearing nonambulatory paraparesis (MFS = 3b), 8 dogs exhibited weight-bearing nonambulatory paraparesis (MFS = 3a), 10 dogs exhibited paraparesis and ataxia (MFS=4), and 2 dogs exhibited spinal hyperesthesia only (MFS = 5).

The median number of rehabilitation visits (including inpatient treatment days) was 9 visits over the 12-week study period (range 1–31). Eleven patients received inpatient rehabilitation associated with their initial presentation. Most patients started with a visit frequency of 1–2 visits per week (range: every other week to 3 visits per week). Visit frequency decreased over the 12 weeks of treatment. Visits ranged from 30 to 60 min.

Ten of the dogs had spinal radiographs prior to the rehabilitation evaluation. One dog had spinal magnetic resonance imaging prior to evaluation which confirmed Hansen Type I disc extrusion at T12-13 and multiple mild disc protrusions between T13 and S1.

Treatment modalities received are summarized in Table 2 and were uniform across MFS groups. Acupuncture and therapeutic exercise were performed on all patients. Electroacupuncture was performed whenever possible due to patient tolerance (21 of 40 patients). Gait training (underwater treadmill or supported land ambulation) was performed with all but 2 patients. The 2 who did not receive gait training were euthanized before this treatment could be implemented. Photobiomodulation therapy was performed on all but 1 patient who had a previously excised soft tissue sarcoma. All but 2 patients received manual therapy as part of their treatment protocol. No explanation was presented in the records for the lack of manual therapy in these cases.

**Table 2:** Number of patients receiving various rehabilitation treatment modalities.

	**Acupuncture**	**Electroacupuncture**	**Therapeutic exercise**	**Gait training**	**Laser**	**Manual**	**HBOT**	**PEMF**	**NMES/TENS**
Yes	40	21	40	38	39	38	10	9	8
No	0	19	0	2	1	2	30	31	32

Less commonly, HBOT, PEMF, and TENS or NMES were included in care (10, 9, and 8 patients, respectively). Hyperbaric oxygen did not become available as a treatment modality in the hospital until late in 2015, midway through the study period. After that time HBOT was offered as part of therapy but occasionally declined by owners due to additional expense. PEMF was not available as a treatment modality in the hospital until 2017. Once available, PEMF was included in visits at no additional cost and used for the majority of patients. NMES was used in cases with poor muscle engagement in conjunction with therapeutic exercise. TENS was used infrequently for patients that did not tolerate electroacupuncture.

Of the 40 dogs in the study, 34 had a positive outcome at 12 weeks after the presentation [85.0%, 95% confidence interval (CI) 70.2–94.2]. Of the 6 dogs without a positive outcome at 12 weeks, 4 had been euthanized and 2 had improved but not yet achieved the functional pet criteria. These 2 continued in rehabilitation past 12 weeks.

The time between the initial development of signs and initiation of rehabilitation is summarized in Table 3. Three of the 4 dogs that were euthanized during the study had started treatment the same day as signs developed, the fourth dog had a 10-day delay in rehabilitation. Of the 2 remaining dogs without a positive outcome at 12 weeks, 1 had no delay of rehabilitation and the other had a 30-day delay. Treatment was initiated within the first 48 h of signs for over half of the dogs in the study.

**Table 3:** Delay between the development of signs and initiation of rehabilitation.

**Delay (days)**	**Number of dogs**
0	16
1–2	6
3–5	10
6–14	4
15–30	4

Outcome at 12 weeks by MFS at presentation is presented in Table 4. Modified Frankel Score at presentation was significantly (*p* < 0.001) higher in the 34 dogs with a positive outcome at 12 weeks (median score 3.5, range 1–5, stands for interquartile range (IQR) 3–4) compared to the 6 dogs that did not have a positive outcome at 12 weeks (median score 0, range 0–2, IQR 0–1). All 27 dogs exhibiting paraparesis, ataxia, or back pain at presentation (MFS 3b or higher), achieved a positive outcome by 12 weeks. Of the 9 dogs exhibiting paraplegia with intact deep nociception at presentation (MFS 1 and 2), 7 dogs (77.8%) had achieved a positive outcome by 12 weeks. Of the dogs in this category with a negative outcome, the dachshund with an MFS of 2 at presentation did not regain independent ambulation. This animal used a cart at home to ambulate and eventually died of other causes. The dachshund with an MFS of 1 at presentation did eventually regain independent ambulation but a positive outcome was not achieved until 26 weeks after presentation. Of the 4 dogs exhibiting paraplegia with persistent loss of deep nociception (MFS 0), none had a positive outcome by 12 weeks, and all 4 were euthanized prior to 12 weeks due to poor condition (1 dog) or a declining condition consistent with progressive spinal cord myelomalacia (3 dogs) (20).

**Table 4:** Outcome at 12 weeks by Modified Frankel Score (MFS) score at presentation.

	**MFS score at presentation**							
**Outcome at 12 weeks**	**0**	**1**	**2**	**3b**	**3a**	**4**	**5**	**Total**
Positive outcome	0	3	4	7	8	10	2	34
Negative outcome	4	1	1	0	0	0	0	6
Lost to follow-up	0	1	2	1	3	2	0	9
								49

**Figure 1:**
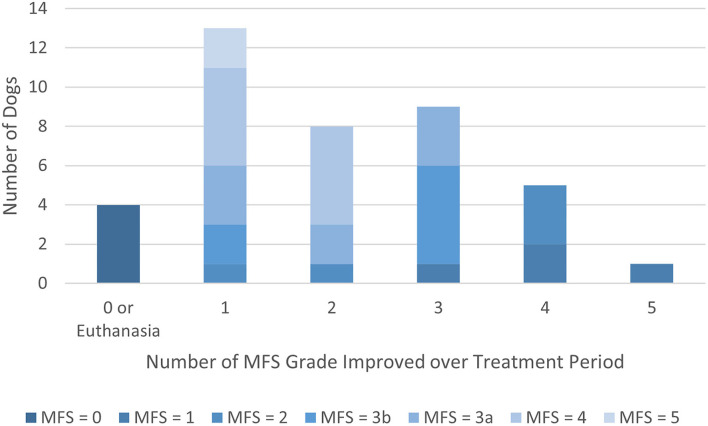
Modified Frankel Score (MFS) grade improvement over treatment period by MFS score at presentation. Shading designates the MFS score at presentation.

As shown in Figure 1, of the 40 dogs with complete records, 36 (90.0%) improved by at least one MFS grade over the course of treatment. Fifteen dogs (37.5%) improved by 3 or more MFS grades. The 4 dogs that did not improve were euthanized during the treatment period.

A review of the records found that 6 of the dachshunds in this study had a history of absent or questionable deep nociception prior to presentation to the rehabilitation service, but deep nociception was noted on initial evaluation. Of these 6, 1 was lost to follow-up, 2 had delayed recovery or incomplete recovery (the negative outcomes from MFS 1 and 2 discussed above), and 3 had a positive outcome by 12 weeks after presentation.

Of the 34 dachshunds with a successful outcome, follow-up records were available beyond the 12-week study period for 27 individuals. The median length of follow-up was 700 days (range: 14–2839 days). The overall rate of recurrence was 14.8% (4/27). Estimated 1- and 2-year recurrence rates were 5.0% (95% CI 0.07–30.5) and 11.3% (95% CI 2.9–38.6), respectively. Four dachshunds had a relapse of signs severe enough to lose functional pet status. One was briefly hospitalized for spinal hyperesthesia and completed another month of rehabilitation to get back to their baseline level of pain control. Two developed weight-bearing paraparesis (MFS 3a) which was resolved with further rehabilitation. One developed paraplegia with intact nociception (MFS 2) and was referred to a neurologist where Hansen Type I intervertebral disc extrusion was confirmed at L1-2 and decompressed *via* hemilaminectomy. The times to recurrence in these 4 dogs were 322, 587, 909, and 1,263 days post-therapy. Overall median time to recurrence could not be estimated in the population due to the low number of events.

## Discussion

In this cohort of dachshunds with presumed Hansen Type I thoracolumbar IVDH, dogs with intact deep nociception, both with and without motor function and superficial nociception, at the time of treatment with nonsurgical rehabilitation had a high rate of return to functional pet status by 12 weeks postinjury. Due to the small number of dogs in each MFS category, further study in a larger cohort is needed to determine whether there are clinically relevant differences in prognosis for return to function between individual MFS categories. None of the 4 dogs lacking deep nociception at the time of treatment achieved functional status in this study. These results align with the surgical literature which finds that dogs with no deep nociception prior to hemilaminectomy are less likely to achieve independent ambulation after surgery (6) and that more severe injuries are associated with a longer time to ambulation postoperatively (7). Additionally, it should be noted that 3 of the patients with an MFS score of 0 at presentation showed signs of progressive neurologic deficits and were presumed to be caused by progressive spinal cord myelomalacia (20).

The results of this study correspond with 3 recent retrospective studies that have evaluated the prognosis for dogs in rehabilitation after T3-L3 hemilaminectomy.

Hady and Schwarz looked at dogs receiving rehabilitation postoperatively. Of the 113 dogs, 23 improved one full MFS. The other 89 did not see a full MFS point improvement. More time in formal rehabilitation and additional sessions on the underwater treadmill significantly increased the chances of a full MFS score improvement (13).

Hogdson et al. evaluated 248 dogs who had undergone hemilaminectomy. They found that more dogs returned to full neurologic function when in-house rehabilitation (passive range of motion, therapeutic exercise, and land or underwater treadmill) was included in postoperative management compared to the control group (33 vs. 9%, respectively). Rehabilitation did not seem to accelerate recovery but was associated with a more complete recovery. Additionally, there was also a lower rate of complications in dogs receiving postoperative rehabilitation (14).

Finally, Jeong et al. evaluated the likelihood of a successful neurologic outcome after surgical decompression of thoracolumbar IVDH between a group receiving postoperative rehabilitation and a control that did not receive postoperative rehabilitation. The group receiving rehabilitation was significantly more likely to have a successful outcome and regained unassisted walking and standing more quickly than the control group. The likelihood of a successful outcome was also associated with the severity of neurologic signs prior to surgery (15). These studies taken together indicate that there is merit for the use of rehabilitation techniques in improving the outcome of spinal cord injury secondary to IVDH.

Research in rodent models has shown the necessity of movement and exercise for functional recovery following spinal cord injury (21). Rats whose hindlimbs were immobilized immediately after spinal cord injury exhibited worsening hindlimb motor function. Even when the animals were allowed to move freely at 8 weeks postinjury, the previously immobilized group never regained the level of motor recovery attained by previously unrestrained controls (22). The above canine studies show the benefit of locomotor gait training in the management of presumed Type I Hansen's IVDH in dogs.

A small, prospective, randomized clinical trial of dogs receiving rehabilitation, rehabilitation and photobiomodulation, or sham treatment after hemilaminectomy found no significant difference between treatment groups in return to function 10 days after surgery. The authors speculated that a longer recovery period may be necessary to note the benefits of postoperative rehabilitation and that rehabilitation may be associated with a higher level of recovery (16). A recent randomized, blinded, prospective clinical trial comparing intensive vs. basic postoperative rehabilitation protocols for 14 days after hemilaminectomy for T3-L3 IVDH found no significant difference between the 30 dogs evaluated in both metrics for ambulation and quadrupedal coordination. The authors postulated that the lack of significant difference may reflect the speed of spontaneous recovery in this group of animals, limiting the benefit of the interventions. Postoperative rehabilitation was deemed safe and given the lack of adverse consequences in the intensive treatment group, a more rigorous rehabilitation regimen may have revealed greater differences between the groups. The authors also speculate that rehabilitation may be more beneficial when targeted toward dogs with more severe signs or with a slower recovery postoperatively (17).

Our results are similar to those of Jadeson published in 1961. In his study, 89.36% of dogs in the rehabilitation group had a good or excellent improvement compared to 85.0% in the current study. For paraplegic dogs, the rates drop to 88.46% for the Jadeson study compared to 77.8% in the current study (18).

In clinical cases where the standard of care, including advanced imaging and surgery, is declined, the veterinarian can advise clients that as long as some nociceptive perception is present 24–48 h after the presentation, a good to excellent prognosis is possible with nonsurgical rehabilitation. Rehabilitation still requires a financial and time commitment from the pet owner but can be a viable alternative to surgical treatment in cases with intact pain sensation.

The 24–48 h window allows time for assessment of true nociceptive abilities. In this study, we found that prognosis worsened if deep nociception was not present at the time of treatment initiation. All 4 dogs with absent deep nociception at the initial rehabilitation evaluation were euthanized during the study period. As noted above, 6 dogs in this study had a record of absent nociception at the time of initial presentation to a veterinarian, although nociception was detected at initial presentation to the rehabilitation service, and 3 of these dogs went on to have a positive outcome at 12 weeks.

This discrepancy may speak to the difficulty of assessing deep nociception during the emergent presentation to a veterinarian, either due to patient temperament in the clinic, severe pain immediately post-IVDH, acute but transient spinal cord swelling and bleeding, or the crudeness of a firm toe pinch to rule in or rule out superficial and deep nociception. Some authors have questioned the reliability of toe pinching as a method for detecting deep nociception in dogs (23). Alternatively, some animals may have experienced a transient loss of deep nociception that had been resolved by the initial rehabilitation evaluation. In the acute phase of spinal cord injury (<24 h), a complete loss of motor and sensory function may be present below the level of an incomplete spinal cord injury due to spinal shock. This would include the loss of deep tendon reflexes and sphincter reflexes. Spinal shock may mask the true degree of injury in the acute period (24). Additionally, rehabilitation veterinarians often employ methods of evoking motor and nociceptive responses in affected limbs using acupuncture or electrical stimulation, which may not be available to clinicians at the initial presentation.

Even cases with a persistent absence of deep nociception may have some chance of recovery with rehabilitation. Joaquim et al. looked at the prognosis for 40 dogs exhibiting signs of severe T3-L3 myelopathy for over 48 h. These dogs received one of 3 treatments: hemilaminectomy alone (10 dogs), hemilaminectomy and electroacupuncture (11 dogs), or electroacupuncture alone (19 dogs). While there was not a significant difference in the proportion of dogs lacking deep nociception in each group prior to treatment, after treatment there were significantly fewer dogs lacking deep nociception in the electroacupuncture-only group. The authors hypothesized that electroacupuncture may help to control the secondary injury cascade through modulation of the immunologic and inflammatory response of the spinal cord (25). Hayashi et al. treated 50 dogs with signs of thoracolumbar IVDH and randomly allocated the dogs to either conservative management (oral steroids, pain medications, activity restriction, bladder management) or electroacupuncture in addition to conservative management. They found that the time to recovery of ambulation was significantly shorter for the group receiving electroacupuncture and that the success rate of achieving unassisted ambulation was higher in dogs receiving electroacupuncture compared to those receiving only conservative management (88.5 and 58.3%, respectively). Three of the 6 dogs in the electroacupuncture group who lacked deep nociception at the start of treatment recovered nociception compared to 1 of 8 dogs in the conservative management-only group (26). This level of recovery in dogs lacking deep nociception is similar to that seen in dogs undergoing surgical intervention in other studies (52.1%) (6). Research on the effects of electroacupuncture after spinal cord injury in rats has found upregulation of the Wnt/β-catenin signaling pathway. This pathway has been shown to be critical in the growth, differentiation, and survival of neurons (27).

If canine patients exhibit a persistent absence of deep nociception (>48 h), and particularly if they show a progression of neurologic signs over 24–48 h, in the face of appropriate pain management and rehabilitation, the prognosis for return to full function is grave.

Of the 34 dachshunds with successful treatment outcomes, we had sufficient records for 27 to assess the likelihood of recurrence. A total of 4 animals (14.8%) exhibited a recurrence of signs severe enough to lose functional pet status, with episodes of worsened neurologic status (MFS 2-3a) or pain episodes requiring hospitalization. Levine et al. reported recurrence of clinical signs in 30.9% of dogs undergoing conservative management without rehabilitation (4), although the smaller sample size in the current study prevents direct comparison to Levine's.

This study is limited by its retrospective nature. An MFS score at presentation must be assigned based on the physical exam findings in the record leaving open the possibility of miscategorization. The authors chose to use the less specific MFS system over the newer and more precise Texas Spinal Cord Injury Score (28), as Van Wie et al. found that the use of the MFS system over the Texas Spinal Cord Injury Score for retrospective studies limits the likelihood of miscategorization (29). There may be some selection bias toward less severe cases in this study if referring veterinarians were more likely to recommend rehabilitation in mild cases. Additionally, there may be a selection bias for owners with a higher commitment to the rehabilitation process. Given the retrospective nature of this study over more than 10 years with 8 different veterinarians, and differences in owner availability, the animals involved did not receive a uniform rehabilitation protocol, although this lack of uniformity would weaken rather than strengthen the significance of our results.

A major limitation of this study is that diagnosis is not confirmed for the majority of patients. Although Hansen's Type I IVDH is a likely cause of T3-L3 myelopathy in dachshunds (1), other etiologies of the disease may be present including neoplasia, vascular events, or infectious and inflammatory disease. Additionally, without imaging it is not possible to determine the severity of disc extrusion or if multiple sites are involved as is often the case for dachshunds with IVDH. This limitation is shared, however, by the veterinarian evaluating these cases at initial presentation when owners decline advanced diagnostics. The authors hope to give a true assessment of the prognosis of nonsurgical rehabilitation in dachshunds, regardless of underlying etiology. We hope that the results of this study will help guide the recommendations of veterinarians faced with seeing these cases in the acute phase when owners are unwilling or unable to pursue advanced imaging and surgery.

Future directions include evaluation of specific rehabilitation treatment protocols with the goal of developing the most cost-effective rehabilitation plan for nonsurgical management of presumed thoracolumbar IVDH in dachshunds. Additionally, long-term monitoring to further illustrate the likelihood of recurrent neurologic deficits or hyperesthesia in animals undergoing nonsurgical rehabilitation compared with animals receiving surgical treatment should be another area of focus. Dachshunds with presumed Hansen Type I IVDH had a good to excellent prognosis for return to status as a functional pet when they presented to a rehabilitation service with sensation or motor function intact. Those patients lacking deep nociception for more than 1–3 days after injury, and especially those with the progression of abnormal neurologic signs, exhibited a grave prognosis. With these results in mind, clinicians should strongly consider rehabilitation as a viable treatment alternative when advanced imaging and surgery are not possible. Additionally, including rehabilitation as a component of conservative management may help to decrease the risk of recurrence of neurologic signs.

## Data Availability Statement

The raw data supporting the conclusions of this article will be made available by the authors, without undue reservation.

## Ethics Statement

Ethical review and approval was not required for the animal study because the study was a retrospective review of medical records. All animals received appropriate care as determined by the treating veterinarian in consultation with the owner of the animal. Written informed consent for participation was not obtained from owners due to the retrospective nature of the study design. Owners consented to treatment plan presented by treating veterinarian.

## Author Contributions

JS conducting the retrospective analysis and authoring the manuscript. JR and BW provided guidance in the planning of the study and the authoring of the manuscript. MG conducted the statistical analysis. All authors contributed to the article and approved the submitted version.

## Conflict of Interest

The authors declare that the research was conducted in the absence of any commercial or financial relationships that could be construed as a potential conflict of interest.

## Publisher's Note

All claims expressed in this article are solely those of the authors and do not necessarily represent those of their affiliated organizations, or those of the publisher, the editors and the reviewers. Any product that may be evaluated in this article, or claim that may be made by its manufacturer, is not guaranteed or endorsed by the publisher.

## Footnotes

^1^StataCorp. 2019. Stata Statistical Software: Release 16. College Station, TX: StataCorp LLC.

## Abbreviations

IVDH: intervertebral disk herniation
HBOT: hyperbaric oxygen therapy
MFS: Modified Frankel Score
NMES: neuromuscular electrical stimulation
PEMF: pulsed electromagnetic field therapy
TENS: transcutaneous electrical nerve stimulation.
